# Safety and Immunogenicity of an mRNA Respiratory Syncytial Virus Vaccine, mRNA-1345, in Healthy and High-Risk Pediatric Populations Aged 2 to 17 Years

**DOI:** 10.1093/ofid/ofag679

**Published:** 2026-10-17

**Authors:** Jennifer Moore, Hui Qian, Sonia K Stoszek, Richard Bennett, Shelly Senders, Scott Striplin, Jennifer L Nayak, Matthew D Snape

**Affiliations:** Moderna Biotech Manufacturing UK Ltd, London, UK; Moderna, Inc., Cambridge, Massachusetts, USA; Moderna, Inc., Cambridge, Massachusetts, USA; Clinical Research Partners, Richmond, Virginia, USA; Senders Pediatrics, South Euclid, Ohio, USA; Velocity Clinical Research, New Orleans, Louisiana, USA; Department of Pediatrics, Division of Pediatric Infectious Diseases, University of Rochester Medical Center, Rochester, New York, USA; Moderna Biotech Manufacturing UK Ltd, London, UK

**Keywords:** healthy, high-risk, messenger RNA, pediatric, respiratory syncytial virus

## Abstract

**Background:**

Respiratory syncytial virus (RSV) is a leading cause of lower respiratory tract infections (LRTIs) in infants and children. No active RSV vaccine is currently available for children. mRNA-1345 is an investigational mRNA-based RSV vaccine encoding the prefusion F (preF) glycoprotein.

**Method:**

This phase 2, randomized, observer-blind study evaluated a single dose of mRNA-1345 in children aged 2 to <18 years who were healthy (2 to <5 years only) or had stable chronic conditions associated with increased RSV risk. Children aged 2 to <5 years received 7.5-, 15-, or 30-µg mRNA-1345 or placebo, whereas children aged 5 to <18 years received 15-, 30-, or 50-µg mRNA-1345. The primary objective was safety and reactogenicity (Part A); the secondary objective was immunogenicity. RSV surveillance was conducted from 6 to 12 months post-immunization in a subset of re-enrolled participants from the 2- to <5-year-old cohort (Part B).

**Results:**

Overall, 346 participants received study vaccination. Across dose levels and age cohorts, mRNA-1345 was well tolerated, with no vaccine-related serious adverse events or discontinuations. mRNA-1345 elicited robust increases in RSV-A and RSV-B neutralizing antibody titers and preF-biased responses by day 29. During Part B surveillance, RSV infections were infrequent, with no severe/hospitalized RSV-LRTIs.

**Conclusions:**

mRNA-1345 demonstrated a favorable safety profile and was immunogenic in a predominantly RSV-seropositive pediatric population. These results support potential further clinical development of this vaccine in children aged >2 years at increased RSV risk, with further evaluation required to characterize the benefit-risk profile in RSV-naïve children.

**Clinical Trials Registration**: NCT06097299

## Teaser

mRNA-1345, an mRNA-based RSV vaccine, was well tolerated and immunogenic in children aged 2 to <18 years, including high-risk groups. Findings support further clinical development of mRNA-1345 in seropositive pediatric patients at risk for more severe RSV disease.

Respiratory syncytial virus (RSV) is the primary cause of lower respiratory tract disease (LRTD) in infants and young children worldwide [1]. RSV significantly impacts those aged <5 years, with an estimated 33.0 million RSV-associated acute lower respiratory infection (LRTI) episodes and 3.6 million RSV-associated acute LRTI hospitalizations globally in 2019 [1]. While the majority of this burden is experienced in children younger than 2 years of age, the incidence of hospitalization due to RSV infection among children aged 2 to <5 years remains substantial, at approximately 2.0–2.8 per 1000 per annum [2]. RSV also represents a considerable disease burden in high-risk children aged <18 years and can lead to hospitalization and death [3]. Despite this burden and over 50 years of research, no active RSV vaccine is available for children [4].

Early vaccine efforts were hindered by adverse outcomes in a 1960s trial of a formalin-inactivated RSV vaccine, which led to enhanced disease upon RSV infection in RSV-naïve infants [4]. Currently, RSV prevention in children relies on passive immunization through maternal vaccination with RSVpreF (Pfizer Inc.) to protect infants aged ≤6 months [5, 6] or monoclonal antibodies—nirsevimab and clesrovimab—for infants during their first RSV season or high-risk children in their second [7].

Advances in antigen design, particularly stabilization of the RSV F protein in the prefusion (preF) conformation, have advanced RSV vaccine development in adults. Currently, the United States Centers for Disease Control and Prevention recommends 3 RSV vaccines for individuals aged ≥75 years and those aged 50–74 years at increased risk of severe RSV disease [8]. Among those recommended is mRNA-1345 (mRESVIA, Moderna, Inc.), an mRNA-based, lipid nanoparticle (LNP)-encapsulated RSV vaccine encoding membrane-anchored RSV F glycoprotein stabilized in the preF conformation. mRNA-1345 has been approved in multiple countries in adults aged ≥60 years and, in some countries, those aged 18–59 years at increased risk for RSV-LRTD [9]. Clinical trials in children are ongoing. In a phase 1 study in RSV-seropositive children aged 12–59 months, mRNA-1345 (15 or 30 μg) was well tolerated, and a single dose increased RSV neutralizing antibody (nAb) titers, with no added benefit from subsequent doses [10]. Here, we report phase 2 data from a study evaluating safety, reactogenicity, and immunogenicity of mRNA-1345 in healthy children (aged 2 to <5 years only) or children at high risk for RSV disease aged 2 to <18 years.

## METHODS

### Study Design and Participants

This phase 2, randomized, observer-blind study (NCT06097299) evaluated the safety, reactogenicity, and immunogenicity of a single injection of mRNA-1345 in children aged 2 to <5 years who were healthy or had stable chronic conditions that increased RSV disease risk and in children aged 5 to <18 years with stable chronic conditions that increased RSV disease risk. Part A evaluated the safety, reactogenicity, and immunogenicity of mRNA-1345 over 6 months; Part B assessed surveillance for respiratory infections and was added following identification of a safety signal in much younger RSV-naïve infants in a separate study (mRNA-1365-P101) [11]. The present study was conducted across 37 centers in the United States between October 24, 2023, and June 27, 2025.

In Part A, Cohort 1 included participants aged 2 to <5 years who were healthy or had a stable chronic condition that increased RSV disease risk, and Cohort 2 included participants aged 5 to <18 years with a stable chronic condition that increased RSV disease risk. Prior RSV infection status was unknown at enrollment. A full list of inclusion/exclusion criteria and relevant chronic conditions is included in the Supplementary Materials.

In Part A, eligible participants were enrolled and randomly assigned via interactive response technology to receive mRNA-1345 (Cohort 1: 7.5, 15, or 30 µg; Cohort 2: 15, 30, or 50 µg) or placebo (Cohort 1 only). In Cohort 1, a placebo group was included to help distinguish common intercurrent illness from vaccine-related adverse events and immune responses following natural RSV infection from vaccine-induced immunity. Details on randomization, blinding, and the rationale for placebo are included in the Supplementary Materials.

In Part B, all participants enrolled and dosed in Part A Cohort 1 (2 to <5 years) were offered the opportunity to re-enroll into the safety follow-up study, in which surveillance for RSV disease was conducted over 6 months (ie, approximately months 6–12 of the study). No study intervention was administered.

The study was conducted in accordance with the protocol, applicable laws, and regulatory requirements; the International Council for Harmonisation Good Clinical Practice guidelines; and the consensus ethical principles derived from international guidelines, including the Declaration of Helsinki and Council for International Organizations of Medical Sciences International Ethical Guidelines. The protocol was approved by the institutional review board (Advarra, Columbia, MD, USA), and written informed consent for study participation was provided for all participants by their parent(s)/legal guardian(s) prior to enrollment.

### Study Vaccine

mRNA-1345 is an LNP formulation comprising a single mRNA sequence encoding RSV preF [12]. Normal saline (0.9% sodium chloride) was used as placebo. All vaccine volumes were 0.5 mL and were administered intramuscularly on day 1.

### Study Objectives

#### Part A

In Part A, the primary objective was to evaluate the safety and reactogenicity of a single mRNA-1345 injection at each of 3 dose levels in Cohort 1 (7.5, 15, and 30 μg) and Cohort 2 (15, 30, and 50 μg). The secondary objective was to evaluate the humoral immunogenicity by RSV-A and RSV-B nAb responses and antigen-specific binding antibody (bAb) responses (preF, postfusion F [postF]) at days 1 and 29 and at 6 months (Cohort 1 only).

#### Part B

In Part B, the primary objective was to evaluate the incidence of RSV-associated respiratory tract disease (RTD) during the 6 months after re-enrollment. The secondary objectives included the safety of a single mRNA-1345 injection administered in Part A through 6 months after re-enrollment.

### Reactogenicity and Safety Assessments

#### Part A

In both Part A cohorts, endpoints included solicited local and systemic adverse reactions (ARs) within 7 days after the study injection, unsolicited adverse events (AEs) through 28 days after the study injection, serious AEs (SAEs), AEs of special interest (AESIs), AEs leading to discontinuation of trial, and medically attended AEs (MAAEs) throughout the study. Additional details are provided in the Supplementary Materials.

#### Part B

In Part B, AESIs and SAEs were recorded from Part B Cohort 1 day 1 until the end-of-study visit. All safety analyses were based on the Part B Cohort 1 Safety Set. Additional details are provided in the Supplementary Materials.

### Immunogenicity Assessments

Blood samples were collected on days 1 and 29 (both Part A cohorts) and month 6 (Cohort 1 only).

Humoral immunogenicity endpoints included RSV-A and RSV-B nAb serum geometric mean titers (GMTs; international units per milliliter [IU/mL] per World Health Organization International Standard for Antiserum to RSV [13]) and RSV preF- and postF-specific bAb serum geometric mean concentrations (GMCs; arbitrary units/mL). Geometric mean fold rises (GMFRs) from baseline were also determined. Serum nAb titers were measured by microneutralization assay and serum bAb concentrations by Luminex assay, as described previously [14].

Cell-mediated immunogenicity (CMI) was planned as an exploratory assessment in Cohort 1 using peripheral blood mononuclear cells collected on days 1 and 29 and month 6 in a subset of Part A participants and optionally 3–24 days after confirmed RSV-LRTD in Part B.

### Surveillance

RSV surveillance was conducted in Part B weekly during RSV season (approximately October 1, 2024, through April 30, 2025) and monthly during RSV off-season. RSV surveillance was not conducted in Part A; however, medically attended respiratory tract infections and hospitalizations were reported as MAAEs and SAEs. Incidence of RSV-RTD, RSV-LRTD, RSV severe LRTD, RSV very severe LRTD, RSV hospitalization, medically attended RSV-RTD (MA-RSV-RTD), MA-RSV-LRTD, MA-RSV severe LRTD, and MA-RSV very severe LRTD from Part B Cohort 1 day 1 through end of study were summarized (Supplementary Table S1). Additional details are in the Supplementary Materials.

### Statistical Analyses

All safety analyses except for solicited local and systemic ARs were conducted using the Safety Set, which included all participants who received vaccination. Solicited ARs were assessed in the Solicited Safety Set, which included all participants in the safety population who contributed any solicited AR data. All immunogenicity analyses were conducted using the per-protocol set, which included all randomly assigned participants who received the planned dose of study intervention, complied with the immunogenicity testing schedule, had baseline and day 29 assessments, and had no major protocol deviations that would impact the immune response. Additional details are in the Supplementary Materials.

No formal statistical hypothesis testing was planned for the study. All statistical analyses were descriptive in nature. GMTs, GMCs, and GMFRs were calculated on log-transformed data with corresponding 95% confidence intervals (CIs). Seroresponse rates were summarized with 2-sided 95% CIs using the Clopper-Pearson method. No multiplicity adjustments were applied. Further details on the statistics conducted can be found in the Supplementary Materials. All statistical analyses were conducted using SAS version 9.4 or higher (SAS Institute Inc., Cary, North Carolina).

## RESULTS

### Participants

In Cohort 1 of Part A, 164 participants were randomized and 162 received the study dose (mRNA-1345 7.5 µg, n = 39; mRNA-1345 15 µg, n = 41; mRNA-1345 30 µg, n = 41; placebo, n = 41; Figure 1); a respective 36 (90.0%), 37 (88.1%), 40 (97.6%), and 35 (85.4%) participants completed the study. In Cohort 2, 185 participants were randomized and 184 received the study dose (mRNA-1345 15 µg, n = 61; mRNA-1345 30 µg, n = 61; mRNA-1345 50 µg, n = 62); a respective 60 (96.4%), 59 (96.7%), and 59 (95.2%) participants completed the study. The most common reason for study discontinuation in both cohorts was loss to follow-up; no participants discontinued due to AEs. Part B included 24 participants each from the mRNA-1345 7.5-µg and 30-µg groups, 25 from the mRNA-1345 15-µg group, and 19 from the placebo group; 1 participant in the mRNA-1345 30-µg group discontinued from the study (lost to follow-up; Figure 1). Median duration of follow-up after vaccination was approximately 180 days in Cohort 1 and 176 days in Cohort 2 and extended to approximately 520 days for participants who re-enrolled in Part B.

**Figure 1.**
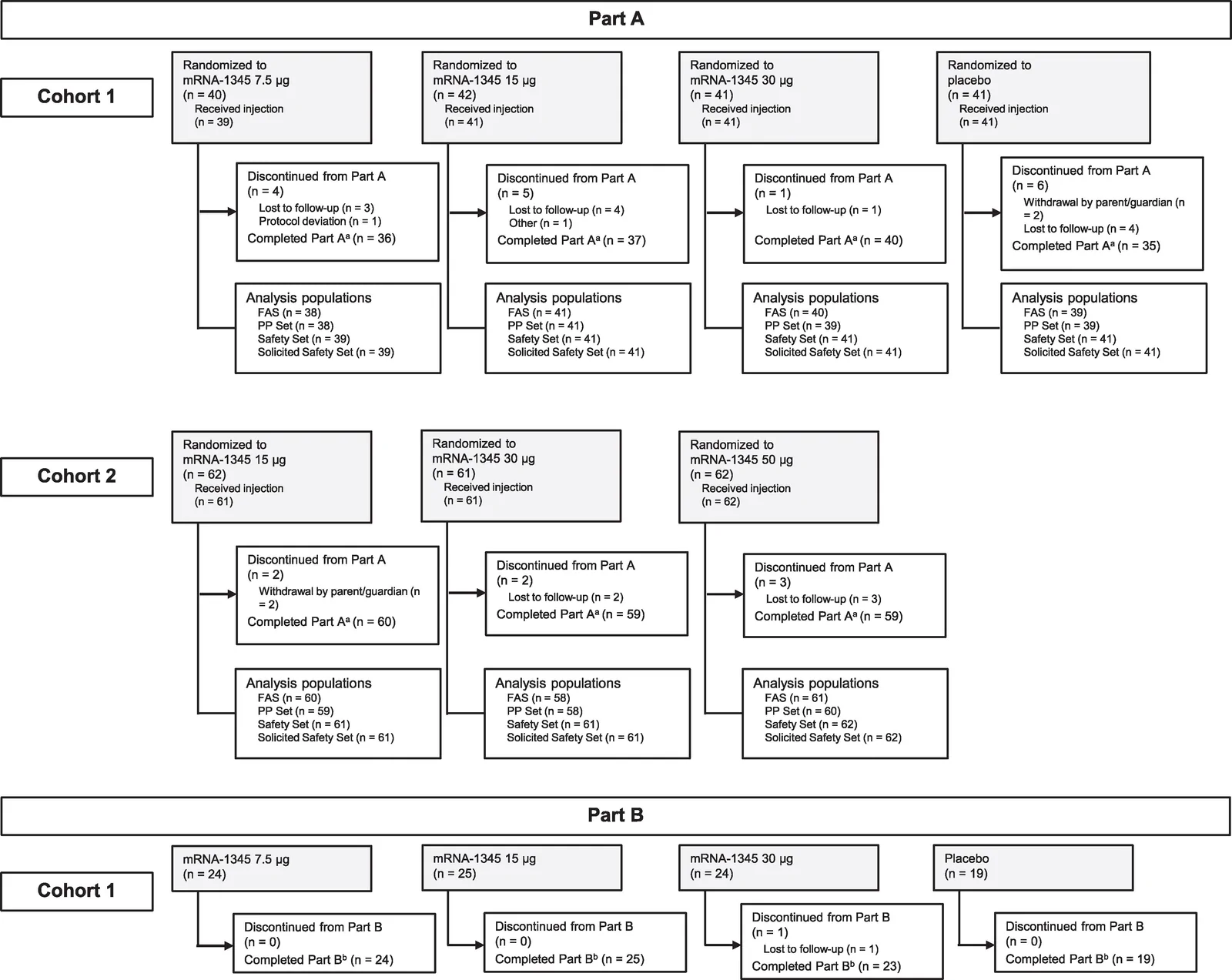
Participant disposition. In Part A Cohort 1, 164 participants were randomly assigned across 3 mRNA-1345 dose levels and placebo groups; in Cohort 2, 185 participants were randomly assigned across 3 mRNA-1345 dose levels. A total of 92 participants from Part A Cohort 1 were re-enrolled into Part B Cohort 1. ^a^Participants were considered to have completed Part A if they completed the Part A final visit at 6 months after the date of the study injection. ^b^Participants were considered to have completed Part B if they completed the last scheduled procedure at the Part B final visit (6 months after Part B Day 1). Abbreviations: FAS, full analysis set; PP, per-protocol.

In the Part A cohorts, participant demographics and baseline characteristics were generally balanced across vaccine groups (Table 1). In Cohort 1, the median age was 3.0 years, 51.8% were female, 68.9% were White, and 35.4% were Hispanic/Latino. In Cohort 2, the median age was 12.0 years, 49.2% were female, 51.9% were White, and 24.9% were Hispanic/Latino. In Cohort 2, approximately 96% of participants had medically treated asthma, with the remainder having other stable chronic conditions associated with increased RSV risk.

**Table 1.** Baseline Demographics and Clinical Characteristics in Parts A and B (Randomization Set)

	Cohort 1: 2 to <5 Years				Cohort 2: 5 to <18 Years		
		mRNA-1345			mRNA-1345		
	Placebo (N = 41)	7.5 µg (N = 40)	15 µg (N = 42)	30 µg (N = 41)	15 µg (N = 62)	30 µg (N = 61)	50 µg (N = 62)
**Part A**							
Age, years							
Median (min, max)	3.0 (2, 4)	3.0 (2, 4)	3.0 (2, 4)	3.0 (2, 4)	12.0 (5, 17)	12.0 (5, 17)	11.5 (5, 17)
Sex, n (%)							
Female	20 (48.8)	22 (55.0)	17 (40.5)	26 (63.4)	33 (53.2)	23 (37.7)	35 (56.5)
Male	21 (51.2)	18 (45.0)	25 (59.5)	15 (36.6)	29 (46.8)	38 (62.3)	27 (43.5)
Race, n (%)							
White	25 (61.0)	26 (65.0)	32 (76.2)	30 (73.2)	38 (61.3)	27 (44.3)	31 (50.0)
Black/African American	14 (34.1)	11 (27.5)	6 (14.3)	8 (19.5)	20 (32.3)	31 (50.8)	28 (45.2)
Asian	1 (2.4)	1 (2.5)	1 (2.4)	2 (4.9)	1 (1.6)	0	1 (1.6)
American Indian or Alaska Native	0	0	0	0	0	0	0
Native Hawaiian or Other Pacific Islander	0	0	0	0	0	0	0
Other	0	0	0	0	0	0	0
Multiple	1 (2.4)	2 (5.0)	3 (7.1)	1 (2.4)	3 (4.8)	3 (4.9)	2 (3.2)
Not reported	0	0	0	0	0	0	0
Unknown	0	0	0	0	0	0	0
Ethnicity, n (%)							
Hispanic or Latino	14 (34.1)	11 (27.5)	14 (33.3)	19 (46.3)	14 (22.6)	17 (27.9)	15 (24.2)
Not Hispanic or Latino	27 (65.9)	29 (72.5)	28 (66.7)	22 (53.7)	48 (77.4)	44 (72.1)	47 (75.8)
Not reported	0	0	0	0	0	0	0
Unknown	0	0	0	0	0	0	0

		mRNA-1345					
	Placebo (N = 19)	7.5 µg (N = 24)	15 µg (N = 25)	30 µg (N = 24)			
**Part B**							
Age at re-enrollment, years							
Median (min, max)	3.0 (2, 5)	4.0 (3, 5)	4.0 (2, 5)	4.0 (3, 5)	NA	NA	NA
Sex, n (%)							
Female	12 (63.2)	13 (54.2)	10 (40.0)	12 (50.0)	NA	NA	NA
Male	7 (36.8)	11 (45.8)	15 (60.0)	12 (50.0)	NA	NA	NA
Race, n (%)					NA	NA	NA
White	14 (73.7)	16 (66.7)	20 (80.0)	17 (70.8)	NA	NA	NA
Black/African American	4 (21.1)	6 (25.0)	2 (8.0)	5 (20.8)	NA	NA	NA
Asian	1 (5.3)	1 (4.2)	1 (4.0)	1 (4.2)	NA	NA	NA
American Indian or Alaska Native	0	0	0	0	NA	NA	NA
Native Hawaiian or Other Pacific Islander	0	0	0	0	NA	NA	NA
Other	0	0	0	0	NA	NA	NA
Multiple	0	1 (4.2)	2 (8.0)	1 (4.2)	NA	NA	NA
Not reported	0	0	0	0	NA	NA	NA
Unknown	0	0	0	0	NA	NA	NA
Ethnicity, n (%)					NA	NA	NA
Hispanic or Latino	9 (47.4)	9 (37.5)	10 (40.0)	13 (54.2)	NA	NA	NA
Not Hispanic or Latino	10 (52.6)	15 (62.5)	15 (60.0)	11 (45.8)	NA	NA	NA
Not reported	0	0	0	0	NA	NA	NA
Unknown	0	0	0	0	NA	NA	NA

Abbreviation: NA, not applicable.

Median participant age in Part B was 4.0 years; 51.1% were female, 72.8% were White, and 44.6% were Hispanic/Latino. Baseline demographics among dose groups were well matched and comparable with Part A Cohort 1.

### Reactogenicity and Safety

#### Part A

In Cohort 1, any solicited local ARs up to 7 days after injection occurred at a numerically higher rate in the mRNA-1345 30-µg group (65.9%) compared with the 15 µg (48.8%), 7.5 µg (41.0%), and placebo (19.5%) groups, and were Grade 1 or 2 in severity; no Grade 3 or 4 events were reported (Figure 2*A*). Injection site pain was the most frequent solicited local AR for all groups (Figure 2*A*). Any solicited systemic ARs occurred at broadly similar rates in all mRNA-1345 groups (43.6%–56.1%) and was lower for the placebo group (36.6%). Most solicited systemic ARs were Grade 1 or 2 (Figure 2*A*). Grade 3 events were reported by 3 participants (mRNA-1345 7.5 µg, fever [n = 1]; mRNA-1345 15 µg, fatigue [n = 1]; mRNA-1345 30 µg, fatigue [n = 1]), and no Grade 4 events were reported. Fatigue was the most frequently reported solicited systemic AR in all groups.

**Figure 2.**
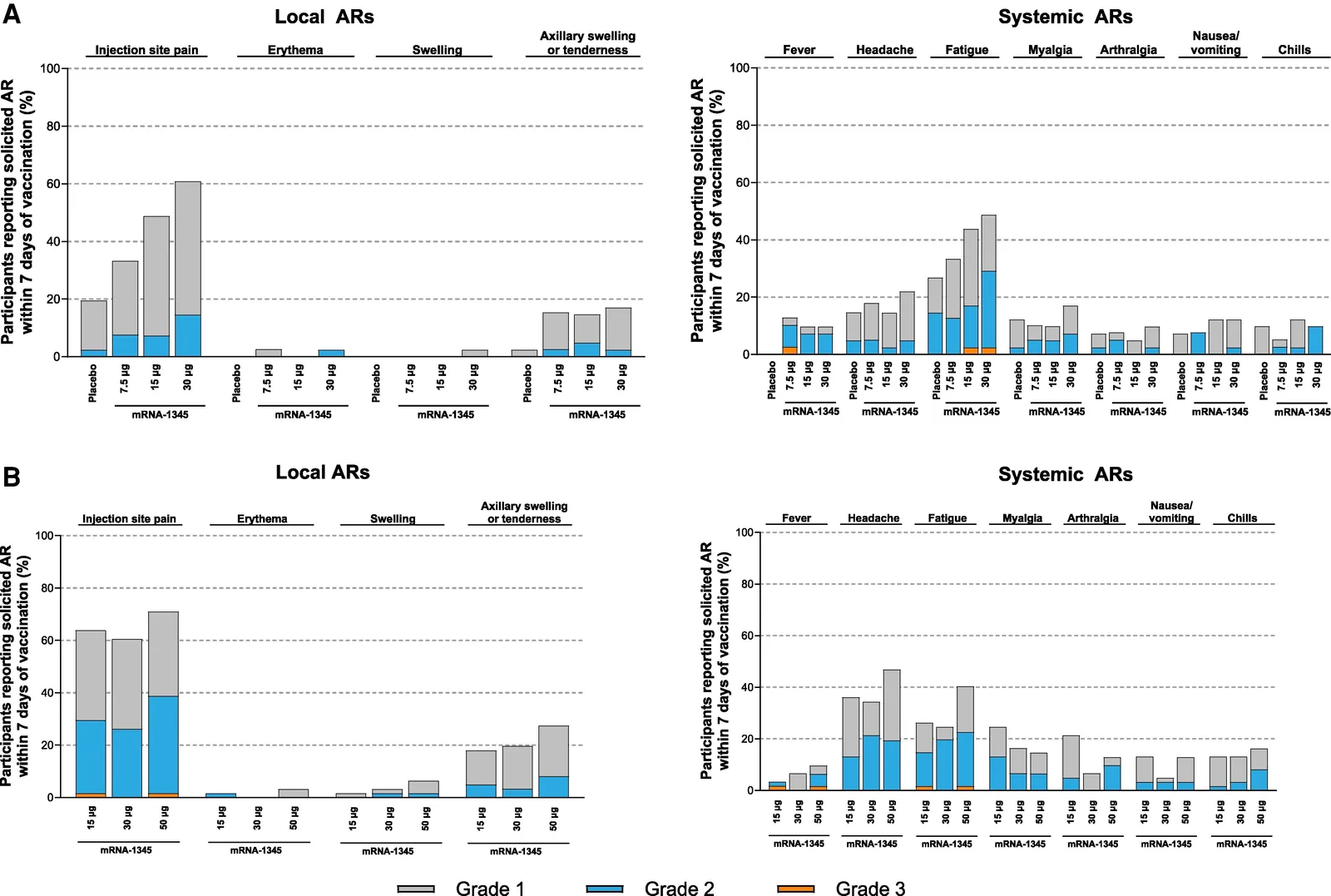
Summary of local and systemic solicited adverse reactions in Cohort 1 (*A*) and Cohort 2 (*B*) within 7 days after vaccination (solicited safety set). Cohort 1 included 41 participants in the placebo group and 39, 41, and 41 participants in the mRNA-1345 7.5-, 15-, and 30-µg dose groups, respectively. Cohort 2 included 61, 61, and 62 participants in the mRNA-1345 15-, 30-, and 50-µg dose groups, respectively. Percentages for individual reaction categories are not additive because participants could report more than one solicited adverse reaction. “Any local AR” and “Any systemic AR” represent participants reporting ≥1 solicited local or systemic adverse reaction, respectively. Abbreviation: AR, adverse reaction.

In Cohort 2, any solicited local ARs occurred at similar rates across all 3 mRNA-1345 dose groups (63.9%–72.6%). Most solicited local ARs were Grade 1 or 2 in severity (Figure 2*B*); Grade 3 injection site pain was reported in both the mRNA-1345 15-µg and 50-µg groups. Injection site pain was the most frequent solicited local AR; the rate was similar across groups. Any solicited systemic ARs occurred at a higher rate in the mRNA-1345 50-µg group (66.1%) compared with the 30-µg (52.5%) and 15-µg (45.9%) groups. Most solicited systemic ARs were Grade 1 or 2 (Figure 2*B*); Grade 3 fever was reported in 2 participants, 1 in the mRNA-1345 15-µg group and 1 in the mRNA-1345 50-µg group; both participants also experienced Grade 3 fatigue. No Grade 4 events were reported. Headache was the most frequently reported solicited systemic AR in all groups (Figure 2*B*).

In Cohort 1, the median (range) duration was 1 (1–5) day (mRNA-1345 7.5 µg and 30 µg), 1.5 (1–6) days (mRNA-1345 15 µg), and 1 (1–6) day (placebo) for solicited local ARs, and 2 (1–6) days (mRNA-1345 7.5 µg and 30 µg), 1 (1–3) day (mRNA-1345 15 µg), and 1 (1–7) day (placebo) for solicited systemic ARs. In Cohort 2, the median (range) duration of solicited local ARs was 2 (1–7) days across all groups. The median duration of solicited systemic ARs was 2.5 days in the 15-µg group, 1 day in the 30-µg group, and 2 days in the 50-µg group.

In both cohorts, rates of reported unsolicited AEs, treatment-related AEs, and MAAEs up to 28 days after injection were similar across groups; no formal statistical comparisons were performed (Supplementary Table S2). No fatal events, study-injection-related SAEs, AESIs (including myocarditis and pericarditis), or events leading to study discontinuation were reported. No SAEs occurred in either cohort in Part A. Up to 28 days after injection in Cohort 2, 1 nonserious medically attended musculoskeletal event (50 µg) and 1 nonserious diarrhea event (50 µg) were considered related to vaccination and resolved without sequelae (Supplementary Table S2).

From 28 days after study injection through Part A end of study, 3 SAEs (1.6%) occurred in the Cohort 2 50-µg group (2 asthma events and 1 post-tonsillectomy hemorrhage). From Part B day 1 through the end of the study, 1 SAE (1.4%) was reported in the 15-μg group of Cohort 1 Part B (influenza). All SAEs were assessed as unrelated to the study injection.

In Part B, where RSV-LRTD was a prespecified AESI, 1 unsolicited AESI of RSV-LRTD was reported in a seropositive participant in the mRNA-1345 7.5-μg group, which was mild in severity and assessed as unrelated to the study injection.

### Immunogenicity

In Cohort 1, all mRNA-1345 dose levels elicited increases in RSV-A and RSV-B GMTs at day 29, with higher GMFRs for RSV-A than for RSV-B and the 30-µg dose generally producing the highest GMFRs; CIs overlapped across doses (Figure 3*A*). At day 29, RSV-A nAb GMFRs from baseline were 0.8 (95% CI, 0.7–1.0) for placebo and 11.9 (95% CI, 7.2–19.4), 5.9 (95% CI, 3.6–9.8), and 12.7 (95% CI, 7.7–20.8) for the mRNA-1345 7.5-, 15-, and 30-µg groups, respectively. At month 6, nAb titers declined but remained above baseline across groups, with the 30-µg dose maintaining the highest GMTs; CIs overlapped across doses. At day 29, RSV-B nAb GMFRs were 0.8 (95% CI, 0.7–1.0) for placebo and 7.3 (95% CI, 4.8–11.1), 4.3 (95% CI, 2.7–6.6), and 8.9 (95% CI, 5.6–14.0) for the 7.5-, 15-, and 30-µg mRNA-1345 groups, respectively. At month 6, GMFRs from baseline for the mRNA-1345 dose groups remained elevated compared with placebo. In Cohort 2, a single dose of mRNA-1345 increased RSV-A and RSV-B nAb titers by day 29 (Figure 3*A*); RSV-A nAb GMFRs from baseline were 8.1 (95% CI, 6.2–10.6), 8.8 (95% CI, 6.3–12.3), and 15.9 (95% CI, 11.9–21.2) for the mRNA-1345 15-, 30-, and 50-µg dose groups, respectively, and RSV-B nAb GMFRs were a respective 4.6 (95% CI, 3.6–6.0), 5.7 (95% CI, 4.2–7.7), and 8.2 (95% CI, 6.5–10.4). Similar trends were observed with bAb responses (Figure 3*B*); across both cohorts and all dose groups, GMFR was higher for preF versus postF. CMI assessments were planned; however, samples were insufficient for analysis.

**Figure 3.**
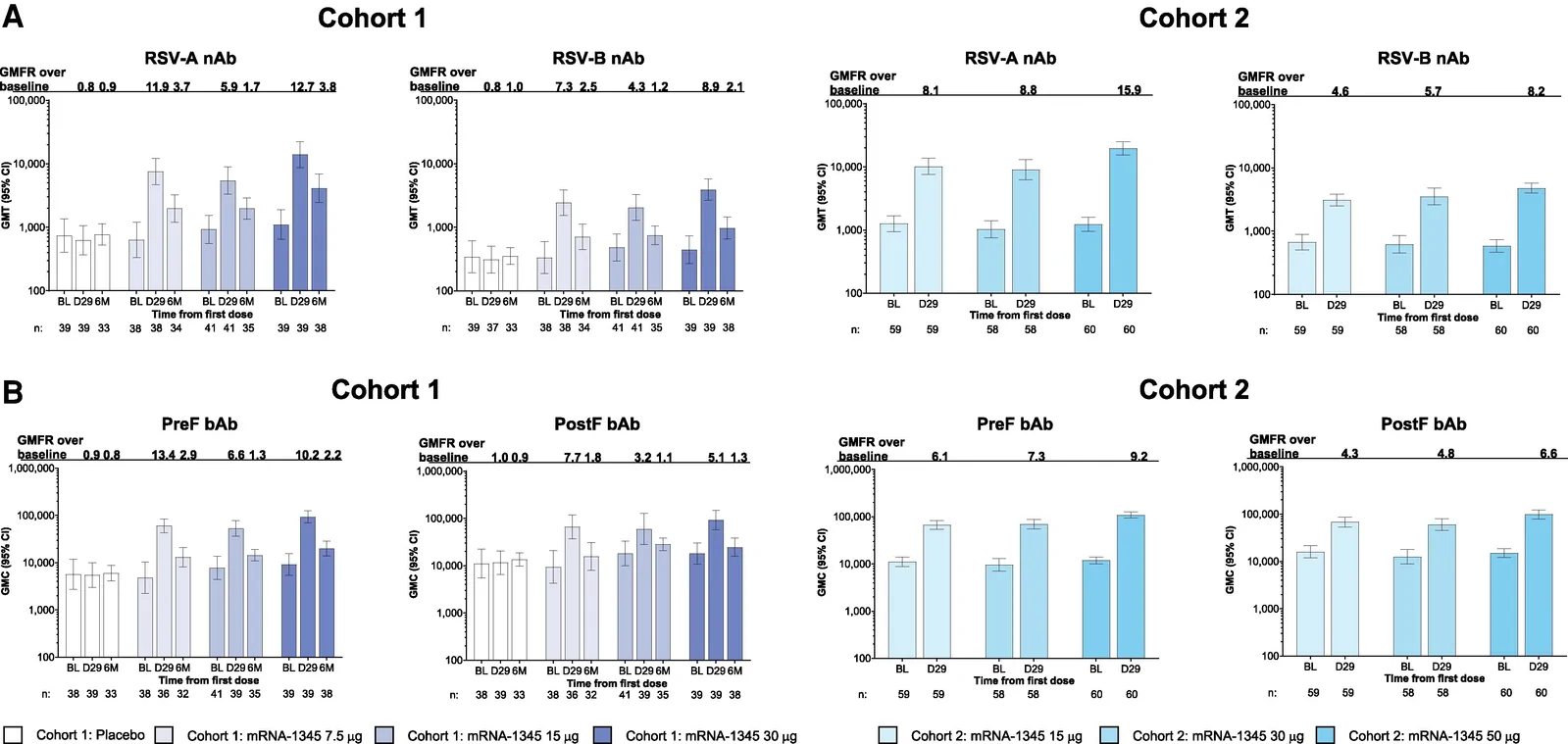
(*A*) Neutralizing and (*B*) binding antibody responses following a single injection with placebo (Cohort 1 only) or mRNA-1345 over time (per-protocol set). The LLOQ and ULOQ for each assay were as follows: RSV-A nAb, 13 and 259061; RSV-B nAb, 10 and 112476; preF bAb, 35 and 580553; postF bAb, 57 and 847551. Abbreviations: bAb, binding antibody; BL, baseline; D, day; GMC, geometric mean concentration; GMFR, geometric mean fold rise; GMT, geometric mean titer; LLOQ, lower limit of quantification; M, month; nAb, neutralizing antibody; PostF, postfusion F; PreF, prefusion F; RSV, respiratory syncytial virus; ULOQ, upper limit of quantification.

### Exploratory Post Hoc Analysis of Baseline RSV Serostatus

The exploratory post hoc analysis assessed participant baseline RSV serostatus. Among the 2 to <5-year-old cohort, only a small proportion (6.4%) were RSV-naïve at baseline (postF IgG ≤200 AU/mL). Based on serologic criteria, 7 mRNA-1345 recipients were considered RSV-naïve at baseline. Of these, 4 demonstrated minimal immunologic responses following vaccination, consistent with lack of prior RSV exposure. None of the 7 participants experienced an RSV SAE or AESI during Part A. The 4 participants considered most likely to be truly RSV-naïve did not enroll in Part B (see Supplementary Materials for additional results).

### Surveillance

Among the 73 mRNA-1345 recipients from Part A Cohort 1 enrolled into Part B, RSV-RTD occurred in 5 (6.8%), all of whom were baseline RSV-experienced: 2/24 (8.3%), 1/25 (4.0%), and 2/24 (8.3%) in the 7.5-, 15-, and 30-μg groups, respectively (Table 2). There were no reports of RSV-related events in the placebo group (n = 19). No RSV LRTD's occurred in the placebo, 15-μg, or 30-μg groups; within the 7.5-μg group, 1 participant (4.2%) had an RSV-LRTD, and 1 (4.2%) had a potential LRTD between Part A completion and re-enrollment into Part B Cohort 1. Both events were mild in severity, assessed as unrelated to the study vaccine, and resolved without sequelae. No severe or very severe RSV-LRTD events and no RSV hospitalizations were reported.

**Table 2.** Summary of RSV Disease Cases in Part B^a^

	Cohort 1: Part B				
		mRNA-1345			
Participants, n (%)^b^	Placebo (N = 19)	7.5 µg (N = 24)	15 µg (N = 25)	30 µg (N = 24)	Total (N = 73)
RSV-RTD	0	2 (8.3)	1 (4.0)	2 (8.3)	5 (6.8)
RSV-LRTD	0	1 (4.2)	0	0	1 (1.4)
RSV severe LRTD	0	0	0	0	0
RSV very severe LRTD	0	0	0	0	0
RSV hospitalization	0	0	0	0	0
Medically attended^c^ RSV-RTD	0	1 (4.2)	1 (4.0)	1 (4.2)	3 (4.1)
Medically attended RSV-LRTD	0	0	0	0	0
Medically attended RSV severe LRTD	0	0	0	0	0
Medically attended RSV very severe LRTD	0	0	0	0	0

Abbreviations: LRTD, lower respiratory tract disease; RSV, respiratory syncytial virus; RTD, respiratory tract disease.

^a^Classification of RSV cases was made by the clinical assessment team, which reviewed all cases of RSV infections to evaluate their severity according to protocol-defined criteria and other clinically relevant criteria (eg, investigator diagnosis and assessment of severity).

^b^Percentages are based on the number of participants in Part B Cohort 1 Safety Set.

^c^A medically attended case is defined as a healthcare encounter due to respiratory tract disease reported under the same unscheduled illness visit folder in the electronic case report form of the RSV-RTD or RSV-LRTD case.

## DISCUSSION

In this phase 2, randomized, observer-blind study in children aged 2 to <18 years, mRNA-1345 was well tolerated across dose levels and elicited strong humoral responses, with no safety concerns raised.

No vaccine-related SAEs, AEs leading to discontinuation, or AESIs were observed. Reactogenicity was predominantly mild to moderate and consistent with prior pediatric evaluations of mRNA-1345 [10].

All dose levels elicited increases in RSV-A and RSV-B nAb GMTs at day 29, with the 30-µg and 50-µg doses producing the highest GMFRs. A similar response was observed for bAbs, and responses resulted in a higher fold rise for preF versus postF. At month 6 in Cohort 1, nAb titers declined but remained above baseline across all dose groups, with the 30-µg dose maintaining the highest GMTs.

Humoral immunity is strongly implicated in protection against RSV disease. Passive immunization strategies have demonstrated prevention of RSV-associated hospitalization in pediatric populations [15–18]. In an immune correlate analysis of the phase 3 P301 trial of mRNA-1345 in older adults, day 29 RSV-A nAb and preF IgG titers were inversely associated with RSV-LRTD [19]. The results supported a correlate of protection, with nAb responses mediating most of the observed vaccine efficacy. These findings provided the scientific rationale for immunobridging to adults aged 18–59 years to support licensure.

The robust humoral immune responses observed here and in prior evaluations of mRNA-1345 in RSV-seropositive children (aged 12–59 months) [11, 20] are therefore reassuring, aligning with studies in older and high-risk adult populations in which immunogenicity and efficacy have been demonstrated [12, 14, 21, 22]. These findings provide context for the antibody responses observed in this study; however, a correlate of protection has not been established in this pediatric population.

A significant proportion of RSV-LRTD and hospitalization occurs in children beyond infancy, with recent US surveillance data indicating that hospitalization rates in children aged 2–4 years (∼2.0–2.8 per 1000) exceed rates in adults aged ≥75 years (∼1.0–1.7 per 1000) [2]. Older children with underlying conditions remain at increased risk of severe outcomes and complications [3]. Active immunization strategies providing protection across pediatric populations therefore remain an unmet medical need.

Independent of this study, a vaccine-associated enhanced respiratory disease signal was identified in RSV-naïve infants aged 5 to <8 months who received mRNA-1345 or mRNA-1365; as a precaution, extended surveillance (Part B) was implemented during the following RSV season [11].

During the surveillance period from 6 to 12 months following immunization of children aged 2 to <5 years (Part B) in this study, RSV-RTD occurred in a small proportion of baseline RSV-experienced participants (6.8%). No severe/hospitalized RSV-LRTD cases were observed. Although the number of confirmed RSV infections was small and the vaccine-to-placebo ratio was 3:1, the mild clinical course of observed cases and absence of hospitalizations does not suggest enhanced disease in this predominantly seropositive population of 2- to <5-year-olds; however, the sample size and low number of seronegative children in the enrolled population limit definitive conclusions. These findings may reflect either immunologic maturation or differences in immune priming, as most children aged ≥2 years have prior RSV exposure, which may influence the quality of vaccine-induced immune responses.

Strengths of this study include its randomized, observer-blind design, evaluation across multiple pediatric age groups, and integration of short-term safety endpoints and additional RSV clinical surveillance in Part B. The inclusion of children at high risk for severe RSV disease enhances the relevance of the findings to real-world pediatric populations. Limitations include the relatively short immunogenicity follow-up period in Cohort 2 (through day 29), which precludes assessment of durability of the humoral immune response, and the lack of a placebo group in Cohort 2. The study was designed to evaluate safety and immunogenicity rather than clinical efficacy. The addition of RSV surveillance in Part B after study initiation provided valuable additional safety follow-up; however, only a subset of participants from Cohort 1 Part A (92/162) re-enrolled into Part B, and the numbers of RSV infections and placebo recipients limit interpretation of the clinical RSV events. The absence of analyzable CMI samples limits characterization of the broader vaccine-induced immune response.

In conclusion, mRNA-1345 was well tolerated in children aged 2 to <18 years, with no vaccine-related safety concerns identified. A single dose elicited strong nAb responses against RSV-A and RSV-B. The surveillance data further support the safety profile of mRNA-1345 in older pediatric populations. Collectively, these findings suggest the potential for further clinical development of mRNA-1345 in older pediatric patients at risk for more severe RSV disease.

## Supplementary Material

ofag679_Supplementary_Data

## Supplementary Data

Supplementary materials are available at *Open Forum Infectious Diseases* online. Consisting of data provided by the authors to benefit the reader, the posted materials are not copyedited and are the sole responsibility of the authors, so questions or comments should be addressed to the corresponding author.
