# Supplementary material for: Safety and Immunogenicity of an mRNA Respiratory Syncytial Virus Vaccine, mRNA-1345, in Healthy and High-Risk Pediatric Populations Aged 2 to 17 Years
Source: Open Forum Infect Dis. 2026 Oct 17;13(11):ofag679. doi: 10.1093/ofid/ofag679 (PMC13728700; doi:10.1093/ofid/ofag679)
Supplement: Supplementary file 1 [file ofag679_supplementary_data.docx]

# Supplementary Methods

**Full inclusion and exclusion criteria**

Inclusion criteria:

*Cohort 1*

Each participant must have met all of the following criteria to enroll:

1. Aged 2 to <5 years at the time the informed consent was signed.
2. Healthy, or with stable chronic conditions increasing the risk of RSV disease, per the clinical judgment of the investigator. Relevant chronic conditions included:
   - Medically treated asthma
   - Cystic fibrosis
   - Chronic respiratory diseases and malformations of the lung
   - Trisomy 21
   - Neuromuscular disease
   - Cerebral palsy
   - Hemodynamically significant or symptomatic congenital heart disease
   - Sickle cell disease without the need for any blood transfusions in the 3 months prior to enrollment
3. Participant’s parent(s)/legally authorized representative(s) provided written informed consent for participation in the study.

*Cohort 2*

Each participant must have met all of the following criteria to enroll:

1. Aged 5 to <18 years at the time the informed consent was signed.
2. Had a stable chronic condition increasing the risk of RSV disease. Relevant chronic conditions included:
   - Medically treated asthma
   - Cystic fibrosis
   - Chronic respiratory diseases and malformations of the lung
   - Trisomy 21
   - Neuromuscular disease
   - Cerebral palsy
   - Hemodynamically significant or symptomatic congenital heart disease
   - Sickle cell disease without the need for any blood transfusions in the 3 months prior to enrollment
3. Contraceptive use by women of childbearing potential should be consistent with local regulations regarding the methods of contraception for those participating in clinical studies.
   - Had a negative pregnancy test at the screening visit and on the day of injection (Day 1)
   - Had practiced adequate contraception or had abstained from all activities that could result in pregnancy for at least 28 days prior to Day 1
   - Had agreed to continue adequate contraception through 90 days following injection. Adequate female contraception was defined as consistent and correct use of a local health authority approved contraceptive method in accordance with the product label
   - Was not currently breastfeeding
4. Participant’s parent(s)/legally authorized representative(s) provided written informed consent for participation in the study. Depending on local regulations, participants aged 16 years and over could self-consent.
5. Verbal or written assent for study participation (according to local practice) or written assent for study participation (if not self-consenting).

Exclusion criteria (both cohorts):

Participants who met any of the following criteria were excluded from the study:

1. Acutely ill or febrile (temperature ≥38.0°C [100.4°F]) within 72 hours prior to or at the screening visit or Day 1. A participant meeting either of these criteria could be rescheduled for enrollment if the event resolved within the 28-day screening window.
2. History of a diagnosis or condition that, in the judgment of the investigator, could affect study assessment or compromise participant safety, specifically:
   - Congenital or acquired immunodeficiency, including human immunodeficiency virus infection
   - Chronic hepatitis or suspected active hepatitis
   - Had a bleeding disorder that is considered a contraindication to intramuscular injection or phlebotomy
   - Clinically significant thrombocytopenia
   - Dermatologic conditions that could affect local solicited adverse reaction (AR) assessments
   - History of allergic or anaphylactic reactions following a vaccination that required medical intervention
   - Active autoimmune disease
   - History of myocarditis, pericarditis, or myopericarditis
   - Confirmed RSV infection within the last 3 months
   - Epilepsy (history of single febrile seizure allowed)
3. Had received or planned to receive any licensed or authorized vaccine ≤14 days prior to the study vaccine injection (Day 1) or planned to receive a licensed or authorized vaccine within 14 days after the study vaccine injection. Approved nonstudy vaccines included in the routine immunization schedule should not be delayed and, if received ≤14 days prior to Day 1, participants could be rescheduled for enrollment.
4. Receipt of:
   - Any prior systemic immunosuppressants or immune-modifying drugs. Short courses (<7 days) of oral corticosteroids were allowed if completed at least 3 months prior to enrollment. Participants could be rescheduled for enrollment if they no longer met this criterion within the screening window. Inhaled, nasal, and topical steroids were allowed, as was topical tacrolimus
   - Intravenous blood products (red cells, platelets, immunoglobulins) within 3 months prior to enrollment
   - RSV monoclonal antibodies within 6 months prior to enrollment
5. Participated in an interventional clinical study within 28 days (6 months for a study assessing a product unlicensed in this age group) prior to the day of enrollment or planned to do so while enrolled in this study.
6. Had a family member or household contact who was an employee of the research center or otherwise involved with the conduct of the study.
7. A child who had been placed under the control or protection of an agency, organization, institution, or entity by the courts, the government, or a government body, acting in accordance with powers conferred on them by laws and regulation (eg, foster care). Did not include a child who was adopted or had an appointed legal guardian.

**Study Design, Randomization, and Blinding**

In Cohort 1, participants were randomized 1:1:1:1 to receive a single injection of mRNA-1345 (7.5 µg, 15 µg, or 30 µg) or placebo (normal saline), and were stratified by age category (40%, aged 2 to <3 years; 60% aged 3 to <5 years). In Cohort 2, participants were randomized 1:1:1 to receive a single injection of mRNA-1345 (15 µg, 30 µg, or 50 µg), and were stratified by age category (50%, aged 5 to <12 years; 50% aged 12 to <18 years). Placebo was used in Cohort 1 but not in Cohort 2, given the higher background rate of reactogenicity-like symptoms (e.g. fever due to intermittent illness) and potential for natural RSV antibody level increases following co-incident infection in younger children, which were less relevant in older participants.

Participants, clinical staff involved in participant assessment, and study sponsor personnel or their designees were blinded to vaccine allocation. Unblinded study personnel who did not participate in any evaluations and assessments handled vaccine dose preparation and administration.

Study conduct was overseen by an unblinded internal safety team and an independent external data and safety monitoring board (DSMB). Emerging safety signals or protocol-defined pause criteria identified during internal safety reviews were escalated to the DSMB for evaluation.

Members of the public were not involved in the design of this trial, data analysis, interpretation of results, or preparation of the manuscript. The study protocol, outcomes, and procedures were developed by the sponsor in accordance with regulatory requirements and clinical trial standards.

**Reactogenicity and Safety Assessments**

In Part A, solicited local (injection site pain, erythema, swelling/induration, and axillary swelling/tenderness) and systemic (headache, fatigue, myalgia, arthralgia, nausea/vomiting, chills, and fever) ARs were recorded by the participant or participant’s parent(s)/legally authorized representative(s) using an electronic diary and assessed using toxicity grading scales [1].

For participants in Part B, physical examinations were performed at Part B Cohort 1 Day 1 visit and whenever participants experienced symptoms of RSV disease (ie, congestion or runny nose for >24 hours, cough, difficulty breathing, or respiratory distress, including wheezing).

**Surveillance**

The primary analysis of the Cohort 1 Part B RSV case classification summary was based on an assessment by an unblinded internal clinical assessment team (CAT) based on the evaluation of all RSV surveillance data collected for each respiratory illness episode (ie, per-protocol assessment). In addition to the protocol-defined criteria, potential RSV-LRTD was recorded, including any cases reported by the study investigator as an unsolicited AE of RSV bronchiolitis, pneumonia, or LRTD that did not meet the per-protocol LRTD definition. Cases were reviewed and classified by the CAT and/or programmatically based on prespecified criteria. Participants underwent nasal swab collection, and samples were sent for RSV polymerase chain reaction testing at GCL-certified central laboratory and, where available, at a local laboratory.

**Statistical Analysis**

Categorical variables are summarized by frequency and percentage, and continuous variables are summarized using descriptive statistics.

A sample size of 60 or 40 participants per group provides approximately 95% or 87% probability, respectively, of observing at least 1 adverse event (AE) at a true incidence rate of 5%, enabling preliminary assessment of reactogenicity for each dose level, with placebo included in Cohort 1 but omitted in Cohort 2 due to the anticipated higher background rate of serious ARs in the youngest age group that could confound tolerability assessment.

For the immunogenicity endpoints, geometric mean titers (GMTs; neutralizing antibodies[nAbs]) and geometric mean concentrations (GMCs; binding antibodies [bAb]) for mRNA-1345 antigen-specific nAbs and bAbs at each timepoint and geometric mean fold rises (GMFRs) of antibody titers at each post-baseline timepoint over pre-injection baseline at Day 1 were calculated. Corresponding 95% CIs were calculated based on the t-distribution of the log-transformed values or the difference in the log-transformed values for GMT and GMFR, respectively, then back transformed to the original scale. For calculation of GMTs, antibody titers below the lower limit of quantification (LLOQ) were replaced by 0.5 × LLOQ and values greater than the upper limit of quantification (ULOQ) were replaced by the ULOQ. Missing values were not imputed.

Classification of RSV cases was performed by the CAT of the sponsor by evaluating all RSV surveillance data collected for each respiratory illness episode. The classification of RSV cases by the CAT was used as the primary analysis of RSV cases.

**Post hoc analysis**

The RSV infection (“naïve” vs “experienced”) status of participants was unknown at enrollment. A post-hoc statistical analysis of children 5 to 23 months of age enrolled in the mRNA-1365-P101 study identified baseline RSV postF-specific bAb concentrations of ≥ 200 AU/mL for children older than 8 months as an indicator of prior RSV infection [2]. This cutoff was applied to the present study.

**Supplementary Results**

**Exploratory post hoc analysis of baseline RSV serostatus**

A post-hoc analysis using postF IgG ≥200 AU/mL identified 10/157 (6.4%) Cohort 1 participants (all aged 2–3 years) as RSV-seronegative at baseline, with postF IgG below the assay LLOQ. Of these, 7 received mRNA-1345 (7.5 µg: 4; 15 µg: 2; 30 µg: 1) and 3 were in the placebo group. Of the 7 mRNA-1345 recipients, 4 showed only minimal increases in postF bAb concentrations at Day 29, consistent with an unprimed immune response, while 3 demonstrated substantial postF bAb increases, suggesting prior exposure or intercurrent RSV infection despite seronegative classification. One of these high responders participated only in Part A; 2 were enrolled in both Part A and Part B. None of the 4 participants with responses consistent with RSV naivety participated in Part B. Corresponding RSV-A and RSV-B nAb and preF bAb patterns supported this distinction.

**Supplementary Table S1.** RSV Case Definitions for Data Analysis

| RSV-RTD | Runny nose OR blocked nose OR cough AND confirmed RSV infection^a^ |
| --- | --- |
| RSV-LRTD | Cough OR difficulty breathing^b^ AND SpO_2_ <95%^c^ OR RR increase^d^ AND confirmed RSV infection^a^ |
| RSV severe LRTD | Meeting the case definition of RSV-LRTD AND SpO_2_ <93%^c^ OR lower chest wall in-drawing |
| RSV very severe LRTD | Meeting the case definition of RSV-LRTD AND SpO_2_ <90%^c^ OR failure to respond/unconscious |
| RSV hospitalization | Confirmed RSV^a,e^ AND hospitalized for acute medical condition^f^ |

BPM, beats per minute; LRTD, lower respiratory tract disease; MA, medically attended; RT-PCR, reverse transcription polymerase chain reaction; RR, respiratory rate; RSV, respiratory syncytial virus; RTD, respiratory tract disease; SpO_2_, blood oxygen saturation.

For each of the case definitions (RSV-RTD, RSV-LRTD, RSV severe-LRTD, and RSV very severe LRTD) an additional category was created to include the subset of participants who met these definitions and attended a health care consultation (MA-RSV-RTD, MA-RSV-LRTD, MA-RSV severe LRTD, and MA-RSV very severe LRTD).

^a^RSV infection confirmed from nasal swab positive by positive RT-PCR or other local laboratory tests.

^b^Based on investigator observation; difficulty breathing included signs of wheezing, stridor, tachypnea, chest in-drawing, or subcostal or intercostal retractions.

^c^For SpO_2_, the lowest stable value monitored was used.

^d^RR increased defined as >38 BPM for 2- to <3-year-olds, >33 BPM for 3- to <4-year-olds, and >29 BPM for ≥4-year-olds per 99^th^ percentiles for age [3].

^e^RSV sampling and testing was based on the medical judgment of a medical practitioner or driven by algorithm.

^f^Hospitalization was defined as a medical decision in which the participant required overnight admission for observation or treatment.

**Supplementary Table S2.** Summary of Unsolicited AEs^a^ in Cohort 1 and Cohort 2

|  | **Cohort 1: 2 to <5 years** | | | | **Cohort 2: 5 to <18 years** | | |
| --- | --- | --- | --- | --- | --- | --- | --- |
|  |  | **mRNA-1345** | | | **mRNA-1345** |  |  |
| **Unsolicited AEs up to 28 days after injection, n (%)^b^** | **Placebo**  **(N = 41)** | **7.5 µg**  **(N = 39)** | **15 µg**  **(N = 41)** | **30 µg**  **(N = 41)** | **15 µg**  **(N = 62)** | **30 µg**  **(N = 61)** | **50 µg**  **(N = 62)** |
| Regardless of relationship to study injection |  |  |  |  |  |  |  |
| All | 9 (22.0) | 9 (23.1) | 3 (7.3) | 10 (24.4) | 11 (18.0) | 5 (8.2) | 6 (9.7) |
| Serious^c^ | 0 | 0 | 0 | 0 | 0 | 0 | 0 |
| Medically-attended | 3 (7.3) | 3 (7.7) | 2 (4.9) | 5 (12.2) | 6 (9.8) | 3 (4.9) | 2 (3.2) |
| Leading to study discontinuation | 0 | 0 | 0 | 0 | 0 | 0 | 0 |
| Severe/≥ grade 3 | 0 | 0 | 0 | 0 | 0 | 0 | 0 |
| Non-serious^d^ | 9 (22.0) | 9 (23.1) | 3 (7.3) | 10 (24.4) | 11 (18.0) | 5 (8.2) | 6 (9.7) |
| Severe or ≥ grade 3^d^ | 0 | 0 | 0 | 0 | 0 | 0 | 0 |
| ≥1 non-serious event^e^ | 9 (22.0) | 9 (23.1) | 3 (7.3) | 10 (24.4) | 11 (18.0) | 5 (8.2) | 6 (9.7) |
| Severe or ≥ grade 3^e^ | 0 | 0 | 0 | 0 | 0 | 0 | 0 |
| Any AESI | 0 | 0 | 0 | 0 | 0 | 0 | 0 |
| Related to study injection |  |  |  |  |  |  |  |
| All | 0 | 1 (2.6) | 0 | 0 | 0 | 0 | 2 (3.2) |
| Serious | 0 | 0 | 0 | 0 | 0 | 0 | 0 |
| Medically-attended^f^ | 0 | 0 | 0 | 0 | 0 | 0 | 1 (1.6) |
| Leading to study discontinuation | 0 | 0 | 0 | 0 | 0 | 0 | 0 |
| Severe/≥ grade 3 | 0 | 0 | 0 | 0 | 0 | 0 | 0 |
| Non-serious^d^ | 0 | 1 (2.6) | 0 | 0 | 0 | 0 | 2 (3.2) |
| Severe or ≥ grade 3^d^ | 0 | 0 | 0 | 0 | 0 | 0 | 0 |
| ≥1 non-serious event^e^ | 0 | 1 (2.6) | 0 | 0 | 0 | 0 | 2 (3.2) |
| Severe or ≥ grade 3^e^ | 0 | 0 | 0 | 0 | 0 | 0 | 0 |
| Any AESI^g^ | 0 | 0 | 0 | 0 | 0 | 0 | 0 |

AE, adverse event; AESI, adverse event of special interest.

^a^An AE is defined as any event not present before exposure to study injection or any event already present that worsens in intensity or frequency after exposure.

^b^Percentages are based on the number of participants in the safety set.

^c^From 28 days after study injection through end of study, 3 serious AEs occurred in 3 participants in Cohort 2 (2 asthma exacerbations and 1 post-tonsillectomy hemorrhage) that were assessed as unrelated to study injection.

^d^For each section, participants who did not report any serious AE are included in the summary of “non-serious AE” and “severe or ≥ grade 3 non-serious AE.”

^e^Participants with at least 1 nonserious AE are included.

^f^In Cohort 2, 1 medically attended AE was considered related to study injection (musculoskeletal chest pain) and was reported in the mRNA-1345 50-µg group approximately 1 day after the study injection and was resolved the same day; the participant had a normal electrocardiogram and negative troponin level.

^g^No fatal events, AESIs (including myocarditis and pericarditis), or events leading to vaccine discontinuation were reported.

**References**

1.	U.S. Department of Health and Human Services, Food and Drug Administration, Center for Biologics Evaluation and Research. Guidance for Industry. Toxicity Grading Scale for Healthy Adult and Adolescent Volunteers Enrolled in Preventive Vaccine Clinical Trials. **2007**. Available at: <https://www.fda.gov/media/73679/download>. Accessed April 14, 2026.

2.	Food and Drug Administration. Review of Investigational RSV (mRNA-1345) and RSV/hMPV (mRNA-1365) Vaccines in Infants and Children < 2 Years. [Presentation to the Vaccines and Related Biological Products Advisory Committee (VRBPAC)]. US Food and Drug Administration; 2024. **2024**. Available at: <https://www.fda.gov/media/184378/download>. Accessed 17 March, 2026.

3.	Fleming S, Thompson M, Stevens R, et al. Normal ranges of heart rate and respiratory rate in children from birth to 18 years of age: a systematic review of observational studies. Lancet **2011**; 377(9770):1011-8.
